# Sickle cell disease in India: a scoping review from a health systems perspective to identify an agenda for research and action

**DOI:** 10.1136/bmjgh-2020-004322

**Published:** 2021-02-18

**Authors:** Vineet Raman, Tanya Seshadri, Sangeetha V Joice, Prashanth N Srinivas

**Affiliations:** 1 Health equity cluster, Institute of Public Health, Bangalore, India; 2 Tribal Health Resource Centre, Vivekananda Tribal Welfare Center, BR Hills, Karnataka, India; 3 Department of Biochemistry, Malabar Medical College and Research Centre, Modakkallur, India

**Keywords:** health systems

## Abstract

**Introduction:**

Sickle cell disease (SCD) disproportionately impacts Adivasi (tribal) communities in India. Current research has focused on epidemiological and biomedical aspects but there has been scarce research on social determinants and health systems aspects. Given its fragmented distribution, resources and programmes have emerged in west and central India. This scoping review seeks to identify geographical and evidence gaps for action on SCD from a health systems lens.

**Methods:**

We followed a scoping review protocol, using Google Scholar and PubMed for published literature. Keywords used included sickle cell anaemia/disease, health systems, tribal and India. We used Google search for grey literature. We compiled a list of 55 records (of which 35 were retained), with about half pertaining directly to India and others relevant to similar settings. Results were organised and analysed using the WHO health systems framework to identify geographical and evidence gaps.

**Results:**

We found substantial literature on biomedical and clinical aspects of SCD but little on the design and implementation of programmes in community and Adivasi-specific contexts as well as on social determinants of SCD. There were regional gaps in knowledge in southern and northeast India. Wherever community-based programmes exist, they have originated in civil society initiatives and relatively limited state-led primary healthcare-based efforts pointing to weak agenda setting.

**Conclusion:**

Both research and action on SCD especially among tribal populations need immediate attention. While geospatial epidemiology has been well understood, gaps remain in context-specific knowledge for action in several parts, as well as evidence gaps across several health system building blocks, including governance and financing of care. Despite publication of a draft policy, delayed adoption and lapses in implementation have limited the response largely to local communities and non-governmental organisations.

**Key questions**

**What is already known?**

- Certain populations (like tribal people) and regions in India are disproportionately affected by sickle cell disease (SCD) but significant knowledge and action gaps persist in developing comprehensive programmes for care for SCD.
- The literature available on SCD focuses on epidemiological or biomedical aspects, fragmented across disciplines and regions with little attention to the aspects of social determinants or health systems.

**What are the new findings?**

- Significant gaps in comprehensiveness of research exist particularly concerning design, implementation or systematic appraisal of quality of care for patients with SCD; this in addition to lack of long-term studies critically limits the availability of evidence of healthcare outcomes in SCD.
- Studies focusing on individual, community and health system blocks were absent; these studies were lacking in terms of coverage from various geographical regions where SCD is reported.
- Most research drew on health services or innovations provided in NGO settings with few studies analysing government health services’ response to SCD at regional/national level.

**What do the new findings imply?**

- Further studies on SCD need to urgently focus on the gaps in geographical coverage of evidence highlighted as well as in comprehensiveness of evidence across individual, population, services and systems levels.
- Research on SCD in India needs to engage with the axes of social inequalities including socioeconomic position, gender, geography and other social vulnerabilities (currently absent) to address the health inequities in SCD.

## Introduction

Being a neglected health problem, the overall research on sickle cell disease (SCD) has also been scarce in India.^1–3^ Furthermore, research on SCD remains fragmented across disciplinary areas, largely from the biomedical disciplines (especially haematology, pathology and genetics), with limited application of a broad, interdependent and dynamic health systems perspective. The WHO defines a health system to include ‘all organisations, people and actions whose primary intent is to promote, restore or maintain health’ with the goal of ‘improving health and health equity in ways that are responsive, financially fair, and make the best or most efficient, use of resources’.^4 5^ There is currently no national or state-led programmes or frameworks outlining a health systems approach towards SCD, although a draft policy notified by the Ministry of Health and Family Welfare exists.^6^ However, in remote tribal population settings, there have been exceptional non-governmental organisation (NGO)-led efforts at responding to the problem with limited coordination with public health services.

Since the early 20th century, various haemoglobinopathies have been described. The prevalence of sickle-cell haemoglobin, the result of an amino acid substitution due to a point mutation in the gene coding for one of the constituent proteins of haemoglobin, was first examined in India by Australian and British pathologists. Cross-sectional prevalence surveys among various communities in India in the 1950s and 1960s documented prevalence particularly among tribal populations with high variation within and across tribal communities,^7^ ranging from as low as 1% to 40%–55%.^8^ Since then, its prevalence is described as being high among particular tribal communities with regionally higher prevalence in several non-tribal yet socially disadvantaged population groups such as other backward classes and scheduled castes.^8^ Recent reviews of SCD are comprehensive^9^ and have attempted to summarise its spatial and population prevalence largely from sporadic cross-sectional surveys. However, the disease’s distribution in socially disadvantaged population groups who already face difficulties of access to health services and inequities necessitates a health systems approach that goes beyond characterising prevalence and clinical features and attempts to identify actionable gaps in our contextual understanding of the disease. The health systems approach is based on a view that health status and healthcare are determined not only by access to clinical and primary healthcare services, but also through wider social determinants of health and through a complex and dynamic interaction across various functions (building blocks) of an ideal health system that requires a transdisciplinary approach towards building strong health systems. In keeping with making research more people centred, a health systems approach foregrounds local context, actors and ‘human agency attributes and values, and is acutely attentive to the local policy and community context’.^10^ Hence, we propose to examine the research available from a health policy and systems research lens to advance an agenda towards action on SCD.

Various frameworks exist for analysing health systems including the six building blocks framework by the WHO: these are service delivery, health workforce, health information systems, access to essential medicines, financing, leadership/governance.^4^ One of the key principles of health systems thinking, however, is the dynamic and interacting nature of these building blocks and the context-specific nature of their interactions.^11^ Furthermore, the social construction and the people-oriented nature of health systems have been importantly highlighted by various researchers.^10 12 13^ The intersection of neglected tropical disease (NTD) control programmes and health services and systems has also been well documented.^14^ Successful NTD control programmes require effective health systems. Wherever specific NTD control programmes can be integrated with health systems, they can serve to both use existing structures and resources and strengthen new functions that can have system-wide positive effects for other disease control programmes as well.^15^ The biomedical framing of SCD and the neglect of wider social inequalities and determinants of health in Africa have been highlighted, but a similar assessment is lacking for the diverse social, geographical and cultural contexts in India.^16^

SCD disproportionately impacts vulnerable tribal communities in India. Despite many studies documenting epidemiological burden among tribal populations^9^ and the feasibility of screening methods in low-resource settings,^17^ several knowledge and action gaps remain in developing comprehensive programmes to provide care for affected communities. SCD has been designated as an NTD, largely because of its burden on the most disadvantaged populations in sub-Saharan Africa and tribal groups in India.^1^ India has the second highest SCD burden in the world^18^ and within India it impacts socially, politically and economically marginalised groups, especially scheduled tribes.^9^ The higher prevalence of SCD among forest-dwelling tribal communities necessitates an urgent need for action from an equity perspective given other drivers of social inequalities among SCD-affected tribal communities.

In this paper, we aim to apply a health systems lens to assess existing research on SCD and identify key gaps in our knowledge of SCD in India, with a particular focus on the need for research and action on SCD among tribal population of India, in order to guide health systems strengthening initiatives for SCD. By bringing a health systems lens onto SCD literature, we hope to steer the SCD research agenda towards evidence that can guide context-specific action on SCD especially among tribal populations in low/limited-resource settings.

## Methods

### Review protocol

A scoping review protocol was developed (online supplemental file 1). The protocol followed the six-step methodological approach to scoping reviews described by Arksey and O’Malley.^19^ Starting with the research question, the relevant studies were identified through database searches. The search results were scanned based on criteria described below. The information from the papers retained was then mapped onto a framework (equivalence to charting the data) and summarised. The review team included researchers with expertise in biochemistry, community health, and health policy and systems research.

10.1136/bmjgh-2020-004322.supp1 Supplementary data

### Review question and scope

The review sought to particularly focus on tribal population. While various labels are applied for tribal population in India, we have chosen the label tribal population to refer to forest-associated communities that are included in statutory lists (the Scheduled Tribe). For a brief overview on the implications of the different labels used in literature to refer to tribal communities, see Srinivas *et al.*^20^ Literature was screened for relevance to SCD based in India or low-resource, tribal population settings as well as literature that assesses one or more building blocks of health systems. Literature was excluded if it pertained to mainly high-income settings, was exclusively epidemiological inquiry without health system components or did not have a substantial focus on health systems in low resource or community settings. Some records from high-income settings were included in our synthesis if they focused on a vulnerable group. While assessing fit of literature, we focused particularly on tribal communities in south India due to relatively limited research and policy attention to south Indian tribal populations given their relatively smaller numbers in south Indian states, when compared with central and northeast Indian regions.^21–23^

### Search strategy

We identified peer-reviewed research papers through searches in PubMed and Google Scholar and supplemented these with technical reports and other grey literature (not peer reviewed) through Google web search. The searches were performed in January and February 2020 with no limit on the dates of publications. Search terms included Boolean queries comprised of the following terms: sickle cell anaemia/disease, tribal community/population/people, indigenous communities, ST/scheduled tribe, vulnerable/marginalized population, Adivasi, health system, treatment, prevention, Karnataka, south India and India (see online supplemental table 1 supporting information for search strategy).

10.1136/bmjgh-2020-004322.supp2 Supplementary data

### Relevance screening and inclusion criteria

**Figure 1:**
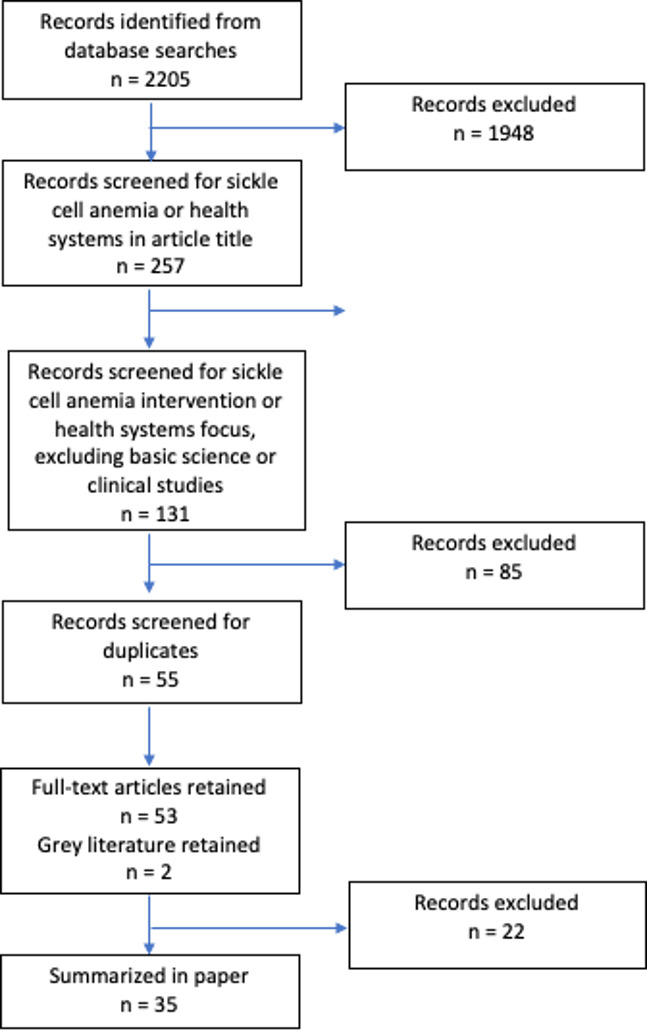
Flow chart on article screening and selection process.

Articles were screened by title to ensure relevance to SCD. The abstracts were then examined to assess relevance for health systems aspects. A total of 131 articles with SCD and/or health systems in their title were identified. Of these, a final list of 35 full-text articles were used in the review (excluded 85 studies that were purely reporting clinical aspects of SCD and 55 duplicates). Each step was performed by the first author and verified by coauthors. The 35 full-text articles were read by at least two authors. A summary of the search term strategy, number of results and screening process is shown below in figure 1. Additional details regarding search terms are available in online supplemental table 1 supporting document. The list of records is available in online supplemental table 2.

10.1136/bmjgh-2020-004322.supp3 Supplementary data

### Conceptual framework

**Figure 2:**
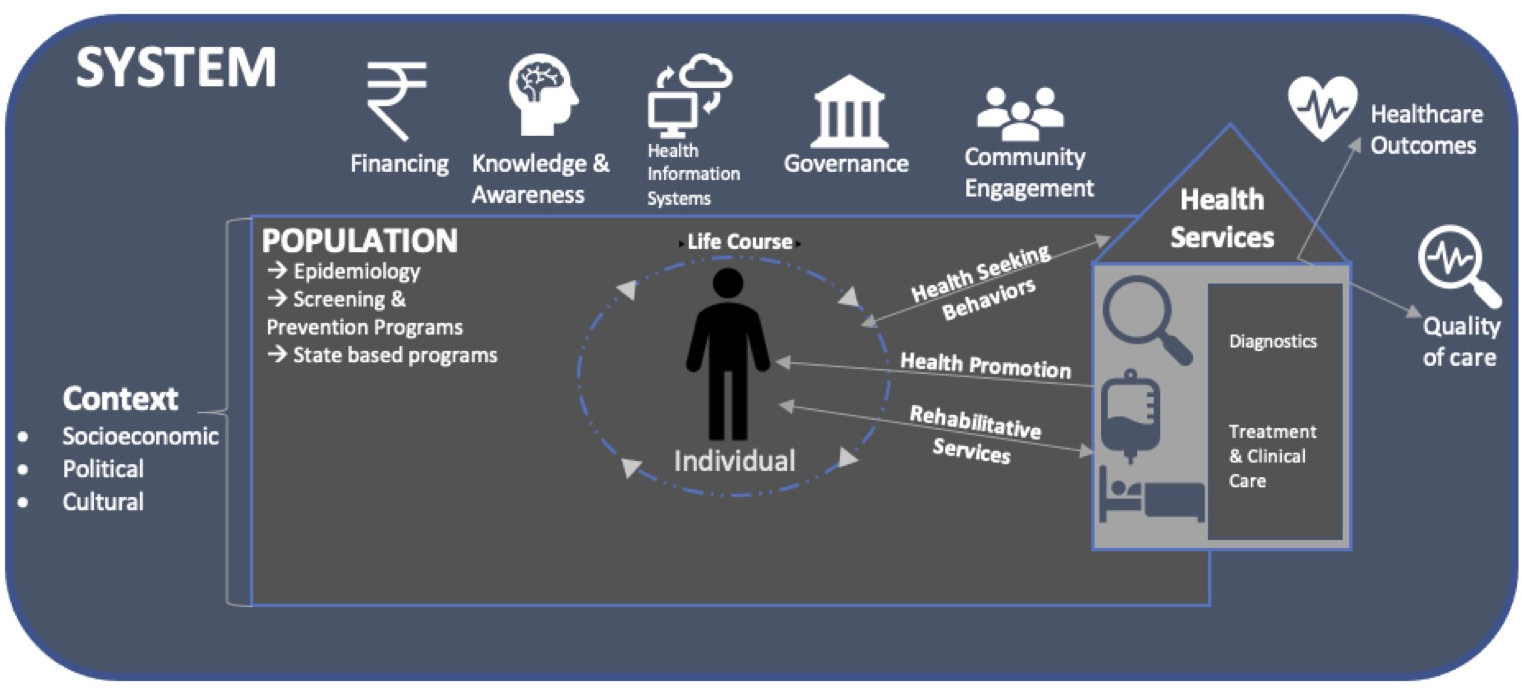
Diagram of conceptual framework for SCD health systems. SCD, sickle cell disease.

In order to organise the evidence identified and identify gaps, there is a need to conceptualise a framework that maps out the elements on which evidence is needed, their relationships and levels at which they operate (individual, populations/community and the wider system). In line with the overall approach in health policy and systems research of grounding research and action agenda with people at the centre,^12^ we iteratively identified themes emerging from the review and from existing health systems frameworks^5 24^ for the purposes of assessing research and action on SCD in Indian settings (see figure 2). To assess comprehensiveness of evidence available for each level and component of the framework, we identified key parameters (online supplemental table 2). We foresee the conceptual framework and these parameters to be an aspirational or ideal set of conditions that helps identify knowledge and evidence gaps to guide the research agenda on SCD in the region. Comprehensiveness was assessed in terms of the parameters defined for each component in online supplemental table 2. For example, to assess comprehensiveness of evidence with respect to life course, we assessed whether the evidence addresses manifestations and illness experience across life course (childhood, neonatal, adult) as well as special categories (pregnant women, elderly). Coverage was measured by assessing geographical coverage across regions.

## Results

### Summary of the literature

Fifty-three peer-reviewed papers along with two of grey literature were retained. Most records were primary literature (n=26), with one-third of the records being secondary literature. Most articles (n=33) were from low/middle-income countries (LMICs), with 25 articles from India. Twelve articles were from the USA and 19 overall were from higher income countries. Most primary authors were medical doctors (n=29) and from LMICs (n=33). Most studies were funded internally by participating organisations/universities. Very few studies were funded by foreign sources (n=7). Governments were the most common funders, whether through grant funding or institutions themselves. Many studies pertained to higher level planning and policy on sickle cell (n=19) and over a dozen detailed interventions in tertiary hospital settings (n=16). Nine studies described community settings. For full details, see online supplemental table 2. Given the importance of screening to detect cases of SCD, service delivery was the most common building block addressed. The second most common building block was access to essential medicines, highlighting the importance of hydroxyurea treatment for those with the sickle phenotype.

### Conceptualising a health systems framework for guiding action on sickle cell anaemia

A health systems response to SCD needs to be organised at multiple levels—individuals, population/community, health system—with associated attributes identified at each level. At the individual level, there is a need to understand SCD effects throughout the life course (age group) and its differential impacts based on various genetic and social attributes (gender, other social identities, including caste and comorbidities and vulnerabilities). At the population/community level, the need is to focus on population health parameters related to epidemiology, access to care and coverage of services/programmes for population screening. At the health systems level, there is a need for identifying evidence related to health services (including aspects related to healthcare delivery such as diagnostics, treatment and clinical care, along with the need for evidence on quality of care provided and healthcare outcomes). At the interface of individuals and health systems too, there is a need for good quality evidence on healthcare-seeking behaviour, health promotion and rehabilitative services to affected individuals. At the health system level, there is a need for evidence on financing, information systems, governance and system-level knowledge/awareness of SCD and its prioritisation. Finally, the importance of various socioeconomic, political and cultural factors influences outcomes at these levels with respect to SCD ought to be understood.

### Synthesis of evidence across health system components

At the individual level, evidence specific to various life stages and health-seeking behaviour was considered. While both comprehensiveness and coverage were good, there is a lack of data from long-term studies addressing specific life stages which are more vulnerable (early infancy and childhood pregnancy). Only two studies examined health-seeking behaviour.^25 26^ Evidence in favour of community-based approaches is available from one specific setting where the involvement of existing trustful relationships between an NGO and the community were crucial in demonstrating effectiveness.^26^

At the population level, epidemiological and population health intervention-related evidence was examined. A recent and as yet, most comprehensive, geospatial analysis is available based on data from 18 states with the majority of the coverage from four states (Gujarat, Maharashtra, Odisha and Chhattisgarh). In terms of population health interventions that are adapted to particular settings, only two studies were found, one of an integrated health network with home visits for education and screening linked to care at a primary care clinic^26^ and another based on volunteer-led screening at village level.^27^

At the health services level, we examined evidence on diagnosis, treatment, rehabilitative services, quality of care and treatment outcomes. This level was the most well studied although studies on rehabilitative services (especially home and community-based approaches) are absent. Lack of long-term studies also limits the availability of fine-scale data on quality of life and healthcare outcomes. The three programmes that are evaluated demonstrate positive outcomes in terms of utilisation and feasibility of community-based interventions for SCD.^26 27^

At the health system level, we examined evidence on financing, information systems, knowledge/awareness, governance and community engagement. Studies on financing and costing of SCD programmes, while scarce, demonstrate the costs of not implementing interventions in terms of the overall National Health Budget.^28^ The cost of blood requirements alone if haemoglobinopathy prevention programmes are not implemented would grow to 19% of the National Health Budget.^28^ In terms of health information systems, the lack of long-term data and registries or limited availability and integration of medical records has been studied with one study focusing on the potential for implementing electronic medical records systems and its implications on SCD.^29^ The knowledge and awareness components were addressed by studies that identified the role for parental education, role of a counsellor and community health worker in improving SCD care in hospital and community settings.^25 26 30 31^ There are no studies that specifically address health systems governance at any level (ranging from local and districts to national), although gaps in governance with respect to ensuring access to SCD medicines have been examined in one study.^31^ There is limited diversity in studying community engagement for SCD with only two studies that examined this.^26 32^

The socioeconomic, political and cultural context of SCD can be studied at multiple levels ranging from individual and households/neighbourhood to policy levels. Socioeconomic context was represented in the epidemiological terms. Only one study examined the political aspects in terms of community ownership at the micro-level. Critical analysis of the macro-level drivers including policies, wider social and political determinants, axes of social inequities including caste, gender, socioeconomic position and other vulnerabilities and how they affect people with SCD was lacking. Only one study examined the role of traditional health systems among one of the indigenous communities in southern Karnataka^33^ despite widespread dependence on traditional medicines among many indigenous and rural communities.^33 34^

## Discussion

Considering the diversity of regional and social contexts from which SCD is reported, there is a need for the future research agenda to specifically address gaps in comprehensiveness of the evidence as well as its socio-geographical coverage. We organised these results in table 1 to illustrate these gaps alongside specific references.

**Table 1:** Synthesis of evidence from papers reviewed organised across levels and health system components along with assessments of their coverage and their comprehensiveness

Level	Health system component	Summary	Cov	Comp
Individual	Life course	Regional and ethnic variations in clinical presentation.^21 25 45^ Early diagnosis and prophylaxis at different life stages.^30 46^ High risk at early childhood and pregnancy.^46 47^ Limited life-course evidence due to lack of long-term follow-up studies.^3 17^		
	Health-seeking behaviours	Delayed or lack of antenatal check-ups and screening especially in tribal populations could increase vulnerability and adverse outcomes.^29^		
Population	Epidemiology	Current estimates based on 249 surveys from 75 sources in 141 spatially unique sites with fragmented coverage across tribal and other high-risk populations. Surveys conducted in 18 of 36 Indian states/union territories with 61% completed in 4 states—Gujarat, Maharashtra, Odisha and Chhattisgarh with scheduled groups being the most studied (171 surveys). Little or no data available in Haryana, Uttarakhand, Uttar Pradesh, Bihar, Central Karnataka and Andhra Pradesh, and the NE states.^18^ Wherever medical records (either electronic or paper based) at health centre exist, such estimates possible but limited experience with EMR.^29^		
	Community-based intervention	Only two studies in one of an integrated health network with home visits for education and screening linked to care at a primary care clinic^26^ and another based on volunteer-led screening at village level.^32^		
Health services	Diagnostics	6 studies reported use of a screening test^17 25–27 30 48^ followed by confirmatory test^26 30^ typically in cross-sectional population studies with 1 long-term follow-up study with follow-up of every 3 months.^31^ 2 papers have recommended longer term cohort study to understand natural history of SCD in India.^3 17^ Community-based screening with home visits linked to primary health centre.^26^ Lack of a systematic SCD screening^17^ although feasibility of newborn screening and continuous care for SCD established in tribal and rural population^31 48^; urgent need to establish uniform gold standard for SCD screening.^49^		
	Treatment	3 studies examining use of combination of hydroxyurea and/or pneumococcal immunisation^26 50 51^ with one establishing the need for counselling and follow-up^51^ and 2 studies on the importance of prophylactic treatment for preventing adverse outcomes.^27 31^ Of these Dave 2019 was an evaluation of a comprehensive SCD programme. Additional treatment with iron supplements in patients with SCD with iron-deficiency anaemia, particularly in tribal adolescent boys and girls.^52 53^ Use of electronic medical records in improving treatment outcomes.^29^ Search for anti-sickling agent continues, including potential ethnopharmacology and bioinformatic-driven approaches.		
	Rehabilitative services	No studies.		
	Quality of care	Although mentioned in one study, it was not specifically evaluated.^26^		
	Healthcare outcomes	Three studies in different parts of India demonstrated Improved utilisation, positive clinical and laboratory outcomes (including decrease in hospitalisation episodes) and acceptable costs of care establishing feasibility of community-based SCD programme.^26 31 49 54^		
Health system	Financing	Only one study^28^ assessed overall financing implications of not implementing prevention programmes and newborn screening for SCD and haemoglobinopathies. One other study^26^ analysed cost details of their community-based intervention.		
	Health information systems	3 studies^26 29 31^ highlight need for health information systems, of which one^29^ specifically evaluates use of EMR in SCD programmes and reports on improved programme efficiency, management and analysis of data at community level. Lack of haemoglobinopathy registry for the country and lack of integrated management and diagnostic facilities for haemoglobinopathies identified as a major deficit.^19^ Giving parents phone for appointment reminders evaluated in one study.^17^		
	Knowledge/awareness	Community-based health worker involvement crucial.^25 26^ Parental education important in SCD life course^30^ and role of counsellor during clinic visits is important for managing complications, improving adherence.^31^		
	Governance	Few studies directly examined health system governance with respect to SCD. One study examined the need for a national prevention programme^28^ and another briefly discussed programme/governance gaps in terms of ensuring access to medicines and vaccines for SCD programmes under the National Rural Health Mission. Involvement of NGO partner was crucial in achieving programme outcomes.^31^		
	Community engagement	Two studies specifically report on community engagement and find positive outcomes in terms of utilisation driven by community engagement.^26 32^		
Context	Socioeconomic	Specific socioeconomic context examined in few studies beyond reporting association with tribal populations, scheduled caste and low socioeconomic groups with high association with illiteracy and poverty.^25^ One study reported on positive effects of community organisation and integration with the healthcare network in an Adivasi setting.^26^		
	Political	Two studies report on the microprocesses around agenda setting for SCD, of which one found community ownership vital to programme success^26^ and in another the request for screening was a felt need from the village.^32^		
	Cultural	The Adivasi Soliga community in southern Karnataka combines traditional medicine and allopathic treatment.^33^		

Cov is short for coverage; comp is short for comprehensiveness. The green colour indicates studies in 4–5 regions, with orange indicating studies in 2–3 regions, and red indicating studies in one region or no studies. Comprehensiveness criteria were established per the criteria established in online supplemental table 2

EMR, electronic medical record; NGO, non-governmental organisation; SCD, sickle cell disease.

### Gaps in comprehensiveness

There were no studies pertaining to design, implementation or effectiveness of rehabilitative services for sickle cell anaemia or systematic appraisal of quality of care for patients with SCD. This is a critical gap that has implications for health systems response to SCD. Even globally, evidence on quality of care and outcomes of SCD is limited to high-income settings where the focus has been on establishing standards of care for adults and children.^35 36^ The lack of long-term studies (such as registries) also limits availability of evidence on healthcare outcomes.^3^ Due to lack of routine data on SCD from government or other health services and the lack of cohort or longitudinal studies involving tribal communities, there is insufficient evidence available related to the life course in Indian settings.

At the systems level, the bias towards studying individuals/communities as opposed to studies focusing on aspects of health policy and systems (governance, policies and financing of care) was conspicuous by their absence. Financing of SCD care in Indian settings is poorly analysed despite increased episodes of hospital admissions, emergency department usage and outpatient visits among patients with SCD being reported from other settings. In a health system with important gaps in achieving universal health coverage, this can impose an unfair burden on poor households further aggravating their socioeconomic hardships. The additional financial and infrastructural costs of caring for SCD in specific geographies need to inform state and regional policy and practice guidelines in order to ensure adequate health systems response, given increased costs of care on primary health centres and local governments with high proportion of patients with SCD.

### Gaps in coverage

Studies on rehabilitative care, quality of care and cultural aspects of SCD were completely absent, whereas studies on governance, financing and community engagement were poor in terms of coverage of different geographical regions from where SCD is reported. Rehabilitative services are one of the four components of primary healthcare but are neglected. Given the varied physical (painful SCD crises among young adults, easy fatigability especially among disadvantaged populations that depend on daily wage physical labour) and psychosocial manifestations of SCD, having access to counselling and social services that can protect patients from socioeconomic consequences is important especially among tribal and disadvantaged community settings. While studies that report on various aspects of the individual life course are good in terms of geographical coverage, population-level studies are mainly drawn from cross-sectional surveys, with limited analysis of routine hospital/healthcare data.

### SCD and tribal populations

There is an overall scarcity of research on health of tribal populations in India,^22^ and the scarcity of evidence for action on SCD on tribal populations is hence not surprising. Given the heterogeneity of the geographical and social contexts of tribal communities, the spatial epidemiological pattern^18^ as well as patterns of health inequalities among tribal populations^20 37^ necessitates a more regional and decentralised research and action agenda for tribal populations. Most studies on SCD tend to draw on health services or innovations provided in civil society/NGO settings (see below) and there were few studies that evaluate or analyse overall government health services or health systems response to SCD at regional/state or national level. The overall agenda setting for research and action on SCD too in terms of design and implementation of interventions is limited to NGO-led initiatives as opposed to state-led ones.^31^ There is scarce literature on the cultural context of SCD in the Adivasi/non-Adivasi landscape and few details of traditional treatments for SCD, except for a single commentary on traditional medicinal preparations used by the Soliga people in southern Karnataka.^33^ In Italy, Colombatti^38^ describes how comprehensive SCD care can be delivered to vulnerable groups and obtain high adherence if linguistic, cultural and social issues are addressed,^38^ echoing the findings of Nimgaonkar and Desai in India.^26 27^ SCD in tribal populations has to be seen in relation to the unfairly higher burden of malnutrition, tuberculosis and malaria, increasing burden of non-communicable diseases and the wider social determinants of their health stemming from poverty, exclusion and historical injustice,^39^ which current literature does not appear to reflect given stronger anchoring within biomedical and epidemiological starting points. Indeed, as pointed out by the Indian government’s expert committee on tribal health, restructuring of health services and systems among tribal populations to allow for greater participation and voice to local communities is vital, and research types that allow documentation of good practices from such initiatives such as implementation research and participatory action research, for instance, were completely lacking with respect to SCD. Without adequate research and implementation sites that allow for this kind of participatory research and action with tribal communities, possible problems with respect to stigma and discrimination may not at all be problematised or investigated but may be important to keep in mind while designing programmes. See for instance the ethical difficulties of implementing population screening programmes that could identify SCD carriers which could fuel stigma and discrimination,^22^ or for instance the public health ethics of population screening and identifying disease without adequate health system preparedness to support them after. This is also a limitation of the analytical framework which relies on building blocks and does not include ethics, discrimination and power imbalances, all of which have contributed to tribal health inequities.^22 39 40^

### Policy response

The need for a policy response in the form of a formal policy document at the national level is an important first step, especially in LMICs.^41^ The current policy response to SCD in India is in the form of a draft policy for prevention and control of haemoglobinopathies (includes thalassemia, SCD and variant haemoglobin conditions) of the central government health ministry^6^ and the guidelines for prevention and control of haemoglobinopathies.^42^ One of the key proposals in the draft policy is to set up centres of excellence for haemoglobinopathies. The proposed centres however do not appear to explicitly focus on rehabilitative services and there is no mention of assessing or facilitating quality of care for patients with SCD. Expectedly, the documents focus on the important themes we identified for which there are studies (laboratory services, human resources, involvement of parent organisations and international collaborations) even if they do not specifically locate these policy options in the current evidence base. Various individual level measures are taken up including prenatal screening, newborn screening and clinical management of SCD. However, due to the lack of adequate geographical coverage of Indian studies, the policy too does not provide any adaptations to the multiple socioeconomic settings in India where affected communities live. Apart from acknowledging the inequities in the disease unfairly affecting economically and geographically disadvantaged communities, the policy response only addresses the curative and biomedical aspects of SCD, which could be a reflection of the lack of health policy and systems research focus and outputs on SCD. Furthermore, the engagement of SCD literature with axes of social inequalities in India including (but not limited to) caste, socioeconomic position, gender and other social vulnerabilities including disability and chronic mental illness is conspicuous by its absence, yet an important input in addressing health inequities in SCD. A recent commentary from the African context has called attention to the value of a social determinants lens in managing sickle cell disorders in sub-Saharan Africa to build a more holistic approach that would promote better health throughout the life course.^16^

In other LMICs (including some countries in Africa), the role of newborn screening, dedicated SCD-specific programmes that include engagement of parents in the care process, education and counselling, and other community-based interventions have been demonstrated to reduce morbidity and mortality.^2 43^ Indeed, regional, national and global collaboration between universities, hospitals and governments across countries with high disease burden is an important step towards creating a systemic environment that could improve diffusion of practices and innovations across these settings.^44^

## Conclusion

Despite availability of adequate evidence on the geospatial epidemiology of SCD, there are gaps in geographical coverage of the evidence across the country, as well as in comprehensiveness of the evidence across individual, population, services and systems levels. Current studies also do not critically engage with the overall health policy and systems environment within which patients with SCD experience their illness. Gaps in evidence at the health policy and systems level also limit solutions at that level and unfairly push the locus of action to individuals and communities. The review has identified key gaps that could inform the research agenda for action on SCD in India.
