# Supplementary material for: Sickle cell disease in India: a scoping review from a health systems perspective to identify an agenda for research and action
Source: BMJ Glob Health. 2021 Feb 18;6(2):e004322. doi: 10.1136/bmjgh-2020-004322 (PMC7896595; doi:10.1136/bmjgh-2020-004322)
Supplement: Supplementary data [file bmjgh-2020-004322supp001.pdf]

S1: Search Terms Supplement

LIST OF KEYWORDS:

- Sickle cell anaemia
- Sickle cell disease

AND

- Tribal community/population/people
- Indigenous communities
- ST/scheduled tribe
- vulnerable/marginalized population
- Adivasi

AND

- Health system
- Treatment
- Prevention

AND

- Karnataka
- South India
- India

For searches, the following exact combinations of keywords were used in the literature review (followed by the number of results generated):

Search Terms	Number of Results
(sickle cell anaemia OR sickle cell disease OR sickle cell anaemia) AND (tribal OR indigenous OR aboriginal or Adivasi OR vulnerable OR scheduled tribe OR marginalized) AND (treatment OR prevention)	139
(sickle cell anaemia OR sickle cell disease OR sickle cell anaemia) AND India) AND (tribal OR indigenous OR aboriginal or Adivasi OR vulnerable OR scheduled tribe OR marginalized) AND (treatment OR opposition)	23
Sickle cell anaemia AND indigenous	33
(sickle cell anaemia OR sickle cell disease OR sickle cell anaemia) AND (south India OR Karnataka OR southern India OR southern Karnataka)	67
(sickle cell anaemia OR sickle cell anaemia OR sickle cell disease) AND (service delivery OR health workforce OR health information systems OR access to essential medicines OR financing OR leadership OR governance) AND (India OR south	62

India OR Karnataka OR south Karnataka OR southern Karnataka)	
(sickle cell anaemia OR sickle cell anaemia OR sickle cell disease) AND (indigenous OR aboriginal OR tribal OR scheduled tribe OR adivasi) AND (service delivery OR health workforce OR health information systems OR access to essential medicines OR financing OR leadership OR governance OR health systems)	18
(sickle cell anaemia OR sickle cell anaemia OR sickle cell disease) AND (indigenous OR aboriginal OR tribal OR scheduled tribe OR adivasi) AND (service delivery OR health workforce OR health information systems OR access to essential medicines OR financing OR leadership OR governance OR health systems) AND (India OR south India OR Karnataka OR south Karnataka OR southern Karnataka)	14
(sickle cell anaemia OR sickle cell anaemia OR sickle cell disease) AND (indigenous OR vulnerable OR marginalized OR aboriginal OR tribal OR scheduled tribe OR adivasi) AND (service delivery OR health workforce OR health information systems OR access to essential medicines OR financing OR leadership OR governance OR health systems)	43
(sickle cell anaemia OR sickle cell disease OR sickle cell anaemia) AND (indigenous OR tribal OR tribe OR aboriginal) AND (India OR Karnataka OR south India OR south Karnataka)	150
