# Supplementary material for: Sickle cell disease in India: a scoping review from a health systems perspective to identify an agenda for research and action
Source: BMJ Glob Health. 2021 Feb 18;6(2):e004322. doi: 10.1136/bmjgh-2020-004322 (PMC7896595; doi:10.1136/bmjgh-2020-004322)
Supplement: Supplementary data [file bmjgh-2020-004322supp002.pdf]

S2: Annotated Conceptual Framework

Level	Components/Factors
Individual	• Life-course - know about the illness - a) how it manifests (symptoms), b) illness experience, c) care needed for different stages of life<br>o Key ones - neonatal, child, adolescent, adult<br>o Special category - pregnant women- maternal, elderly<br>• Health seeking behaviours:<br>o Seeking care in outreach programs - screening programs, access to outreach<br>o Care seeking at facilities for prevention, treatment and rehabilitation, and complications<br>o Self-care + self-reliance
Population	• Epidemiology<br>o Prevalence (disease burden) and estimates (quality of life)<br>o Associated risk factors<br>▪ Individual level<br>▪ Population level<br>o Coverage - whether populations have been studied, gaps if any<br>o Access to care<br>• Community based intervention - anything involving a health outside a health facility<br>o Awareness (e.g. a play), health delivery (health promotion & outreach)<br>o Implemented by any government<br>o Population driven activities for a community
Health Services	• Diagnostics- point of care<br>o Early detection<br>• Treatment + clinical care<br>• Rehabilitative services<br>• Quality of Care - evidence on high admissions due to co-morbidities – a gap in India<br>• Healthcare outcomes<br>• Knowledge/awareness
Health System	• Financing<br>o Funding allocation for programs<br>o Insurance (state/private)<br>▪ Completeness of coverage<br>o Out of pocket payments<br>• Health information systems<br>o Registries<br>o Long-term monitoring/surveillance<br>• Governance<br>o Who is leading the care/financing of SCD - NGO and State interventions have papers, private orgs??

	○ Policy - state/national level<br>• Community engagement - strategies for including ppl in treatment, screening<br>○ Multiple ways - patient support groups
Context	Any studies that use this lens:<br>• Socioeconomic - inequalities in care/coverage of all of the above<br>• Political - Adivasi, social action by tribal groups on SCD care seeking, community organization for a goal<br>• Cultural - how does SC pan out in Adviasi/non-Adivasi landscape, traditional medicine
